# Rapid Generation of In-House Serological Assays Is Comparable to Commercial Kits Critical for Early Response to Pandemics: A Case With SARS-CoV-2

**DOI:** 10.3389/fmed.2022.864972

**Published:** 2022-05-06

**Authors:** Heidi Auerswald, Chanreaksmey Eng, Sokchea Lay, Saraden In, Sokchea Eng, Hoa Thi My Vo, Charya Sith, Sokleaph Cheng, Gauthier Delvallez, Vann Mich, Ngy Meng, Ly Sovann, Kraing Sidonn, Jessica Vanhomwegen, Tineke Cantaert, Philippe Dussart, Veasna Duong, Erik A. Karlsson

**Affiliations:** ^1^Virology Unit, Institut Pasteur du Cambodge, Pasteur Network, Phnom Penh, Cambodia; ^2^Immunology Unit, Institut Pasteur du Cambodge, Pasteur Network, Phnom Penh, Cambodia; ^3^Medical Biology Laboratory, Institut Pasteur du Cambodge, Pasteur Network, Phnom Penh, Cambodia; ^4^Khmer–Soviet Friendship Hospital, Ministry of Health, Phnom Penh, Cambodia; ^5^Communicable Disease Control Department, Ministry of Health, Phnom Penh, Cambodia; ^6^Environment and Infectious Risks Unit, Institut Pasteur, Paris, France; ^7^Institut Pasteur de Madagascar, Pasteur Network, Antananarivo, Madagascar

**Keywords:** SARS-CoV-2, serology, ELISA, PRNT, immunoassay

## Abstract

**Introduction:**

Accurate and sensitive measurement of antibodies is critical to assess the prevalence of infection, especially asymptomatic infection, and to analyze the immune response to vaccination during outbreaks and pandemics. A broad variety of commercial and in-house serological assays are available to cater to different laboratory requirements; however direct comparison is necessary to understand utility.

**Materials and Methods:**

We investigate the performance of six serological methods against SARS-CoV-2 to determine the antibody profile of 250 serum samples, including 234 RT-PCR-confirmed SARS-CoV-2 cases, the majority with asymptomatic presentation (87.2%) at 1–51 days post laboratory diagnosis. First, we compare to the performance of two in-house antibody assays: (i) an in-house IgG ELISA, utilizing UV-inactivated virus, and (ii) a live-virus neutralization assay (PRNT) using the same Cambodian isolate as the ELISA. In-house assays are then compared to standardized commercial anti-SARS-CoV-2 electrochemiluminescence immunoassays (Elecsys ECLIAs, Roche Diagnostics; targeting anti-N and anti-S antibodies) along with a flow cytometry based assay (FACS) that measures IgM and IgG against spike (S) protein and a multiplex microsphere-based immunoassay (MIA) determining the antibodies against various spike and nucleoprotein (N) antigens of SARS-CoV-2 and other coronaviruses (SARS-CoV-1, MERS-CoV, hCoVs 229E, NL63, HKU1).

**Results:**

Overall, specificity of assays was 100%, except for the anti-S IgM flow cytometry based assay (96.2%), and the in-house IgG ELISA (94.2%). Sensitivity ranged from 97.3% for the anti-S ECLIA down to 76.3% for the anti-S IgG flow cytometry based assay. PRNT and in-house IgG ELISA performed similarly well when compared to the commercial ECLIA: sensitivity of ELISA and PRNT was 94.7 and 91.1%, respectively, compared to S- and N-targeting ECLIA with 97.3 and 96.8%, respectively. The MIA revealed cross-reactivity of antibodies from SARS-CoV-2-infected patients to the nucleocapsid of SARS-CoV-1, and the spike S1 domain of HKU1.

**Conclusion:**

In-house serological assays, especially ELISA and PRNT, perform similarly to commercial assays, a critical factor in pandemic response. Selection of suitable immunoassays should be made based on available resources and diagnostic needs.

## Introduction

The global coronavirus disease 2019 (COVID-19) pandemic has emphasized the need for rapid development of assays, especially for early response and outbreak control. Global disruption of trade and value chains, requirements for clinical evaluation and approval, and limited access to virus isolates made establishment of in-house methodologies crucial for early response while the commercial establishment was able to catch up to global demand. Assays to detect antibodies against severe acute respiratory syndrome (SARS) coronavirus (CoV) 2 (SARS-CoV-2) are essential to determine immunity, either upon potential natural exposure (1) or post vaccination (2). Therefore, use of these assays proved critical for case determination, tracking outbreaks, and documentation of vaccination success, especially during the initial phases of the COVID-19 pandemic. Due to high demand in testing worldwide during a pandemic, daily challenges in implementation of necessary serological assays may require in-house solutions that can vary between small-scale research laboratories to fully automated large clinical laboratories. Having a pressing need for a serological assay to monitor SARS-CoV-2 infection and immunity, and having the laboratory resources to employ such an assay, we developed two in-house solutions very early in the pandemic using whole virus isolate until a broad range of different immunoassays were available, including commercial assays that can catering to the available resources, skills, and outcome needs in various facilities (3, 4). As such, the performance between rapid, in-house solutions and commercially available assays needed to be assessed.

In general, SARS-CoV-2-infected individuals seroconvert in the second week post onset of symptoms, with a nearly simultaneous appearance of IgM and IgG (5–7). Most serological assays target the nucleocapsid protein (N), encapsulating the viral RNA genome, and/or the spike (S) protein (3), which is embedded in the virion envelope. The S protein is divided into two main parts (8): the N-terminal S1 domain harboring the receptor-binding domain (RBD) for Angiotensin-converting enzyme 2 (ACE2), and the C-terminal S2 domain contains the fusion peptide mediating the membrane fusion with the target cells. An in-house ELISA utilizes inactivated virus and therefore should be able to detect IgG antibodies directed against all accessible SARS-CoV-2 virion surface epitopes, not only anti-S antibodies. Furthermore, PRNT measures functional antibodies (independent of class) that not only bind to live virions but also neutralize functionality to effectively infect susceptible cells.

In this study, the performance of in-house developed IgG ELISA, using UV-inactivated full-virus antigen from a local Cambodian SARS-CoV-2 isolate, and plaque reduction neutralization test (PRNT) utilizing the same viral isolate as the ELISA were compared then assessed against (i) clinically validated ECLIA from Roche (using either N or S antigen), (ii) flow cytometry based assays (9–12) detecting IgM and IgG binding to S-expressing cells (9–12), and (iii) to a multiplex microsphere-based immunoassay (MIA) using commercially available, recombinant SARS-CoV-1, and−2, MERS-CoV-V and hCoV antigens.

## Materials and Methods

### Specimen Collection

Blood samples were collected opportunistically between January 2020 to October 2020 from hospitals in Phnom Penh including Khmer-Soviet Friendship hospital, during outbreak investigation, and from various provinces for surveillance purposes. A combined oro-nasopharyngeal swab was taken for molecular SARS-CoV-2 detection by RT-PCR. For positive cases, follow-up swabs were taken until two consecutive negative RT-PCR results within 48 h were achieved. During the course of this molecular follow-up, blood samples were taken for routine hemato-biochemical tests at the treatment center/hospital. These samples were sent to the clinical laboratory and left-over blood was used for this project. Blood samples were taken between the actual day of laboratory diagnosis (D0) and 51 days after laboratory diagnosis.

### Ethical Approval

The use of left-over blood samples was approved by National Ethical Committee for Human Research (No. 206 NECHR). Patient's information was anonymized prior to the analysis.

### Cells and Viruses

VeroE6 (ATCC CRL−1586) and Vero (ATCC CCL-81) cells were maintained in Dulbecco's modified Eagle medium (DMEM; Sigma-Aldrich, ST. Louis, MO, USA) supplemented with 10% fetal bovine serum (FBS; Gibco, Gaithersburg, MD, USA), and 100 U/mL penicillin and 100 g/mL streptomycin (Gibco) at 37°C and 5% CO_2_ atmosphere. Antigen production for ELISA and PRNT itself were performed under BSL-3 conditions with live-virus of a Cambodian SARS-CoV-2 isolate designated hCoV-19/Cambodia/1775/2020 (GISAID: EPI_ISL_956384) belonging to the Wuhan lineage. SARS-CoV-2 was propagated in Vero cells infected with an MOI of 0.1 for 1 h at 37°C and 5% CO_2_. Afterwards the inoculum was replaced by DMEM containing 5% FBS, 100 U/mL penicillin and 100 g/mL streptomycin, and 0.25 μg/mL amphotericin B (Gibco). Virus was harvested 2 days after inoculating the cells by centrifugation of the culture supernatant. Titers of viral stocks previously stored at −80°C were determined by plaque assay performed on VeroE6 cells in 48-well plates with 2^*^10^4^ cells/well.

### SARS-CoV-2 Molecular Detection (RT-PCR)

Molecular detection of SARS-CoV-2 in combined oro-nasopharyngeal swabs was performed as previously described (13). Briefly, RNA was extracted with the QIAamp Viral RNA Mini Kit (Qiagen) and real-time RT-PCR assays for SARS-CoV-2 RNA detection were performed by using primers and probes for E and RdRp gene from Charité Virology (Berlin, Germany) (14).

### In-House Enzyme-Linked Immunoassay (ELISA)

The in-house developed ELISA uses UV-inactivated SARS-CoV-2 and allows the detection of IgG antibodies. Virus was produced as described above. Upon harvesting the virus-containing culture supernatant, viral particles were precipitated by adding 40% polyethylene glycole (PEG) 8000 (Sigma-Aldrich) in 1 × PBS to achieve 8% PEG end concentration. The precipitation was carried out overnight at 4°C. The virus was harvested the following day by centrifugation at 1,500 rpm for 1 h. The virus-containing pellet was dissolved in ELISA coating buffer (2.9 mM sodium carbonate, 7.14 mM sodium bicarbonate; Sigma-Aldrich; pH 9.6). The virus solution was inactivated by exposure to UV light for 15 min at RT (in a thin layer within an open 6-well plate). The efficacy of inactivation was determined by plaque assay (13). The protein concentration was determined by Bradford reaction (Protein assay dye, BioRad, Cercules, CA, US), and the concentration was adjusted to 100 μg/mL. Afterwards, 96-well polysorb plates (Nunc, Roskilde, Denmark) were coated with 100 μL/well (10 μg/well) overnight at 4°C. The next day, plates were washed three times with 1 × PBST [1 × PBS containing 0.05 (v/v) Tween 20, Sigma-Aldrich]. Afterwards, the plates were incubated firstly with blocking solution [5% (w/v) low fat milk powder in 1 × PBST] for 1 h, and secondly with patient samples in duplicate (1:40 diluted in blocking solution) for 2 h. After washing three times, plates were incubated with anti-human IgG HRP conjugate (KPL, Gaithersburg, MD, USA; diluted 1:1,000 in blocking solution) for 1 h, and after washing three times with PBST and afterwards three times with PBS, the plates were lastly incubated with ABTS substrate (KPL) for 20 min. The colorimetric reaction was measured at 405 nm with a Multiskan FC microplate photometer (Thermo Fisher Scientific, Walham, MA, USA). The threshold for positivity was set at OD_405_ ≥ 1. The assay was calibrated using the NIBSC plasma sets 20/118 and 20/130 (Supplementary Table 3).

### Plaque Reduction Neutralization Test (PRNT)

The PRNT is the gold standard assay detecting neutralizing antibodies against SARS-CoV-2. VeroE6 cells were seeded in 24-well plates at 1.5 × 105 cell/well. Patients' sera were 2-fold diluted and incubated with SARS-CoV-2 (Cambodian isolate, GISAID: EPI_ISL_956384, ~50 PFU/well) for 1 h at 37°C, 5% CO_2_. The virus-serum dilutions were incubated to allow antibody-mediated neutralization of the virus before transferring the mixtures onto the cell monolayers for 30 min. Next, the inoculation mixtures were replaced by a semi-solid overlay medium containing 1% carboxymethyl cellulose (CMC; Sigma-Aldrich) dissolved in DMEM, 5% FBS, 100 U/mL penicillin and 100 g/mL streptomycin. After 3 days of incubation at 37°C and 5% CO_2_, cells were fixed and the virus inactivated by treatment with 4% formaldehyde (General Drugs House Co. Ltd., Bangkok, Thailand) in 1 × PBS (Sigma-Aldrich) for 30 min. This was replaced with a staining solution containing 1% crystal violet (Sigma-Aldrich), 4% formaldehyde, 1% Methanol (Merck, Darmstadt, Germany) and 20% ethanol (Merck). After 20 min the staining solution was removed, plates were carefully washed with water and then dried at RT. Infection events appear as unstained plaques and were counted by naked eye. The amount of neutralizing antibodies was expressed as the reciprocal serum dilution that induced 50% reduction of infection (PRNT_50_) compared to the positive control (virus only) and was calculated by log probit regression analysis (SPSS for Windows, Version 16.0, SPSS Inc., Chicago, IL, USA). PRNT_50_ titers below 10 were considered negative. The assay was evaluated using the NIBSC plasma sets 20/118 and 20/130 (Supplementary Table 3), and is currently in use for the serological investigation of surveillance samples at the COVID-19 WHO Global Referral Laboratory at Institut Pasteur in Cambodia.

### Commercial Electrochemiluminescent Immunoassays (ECLIAs)

The Elecsys immunoassays are automatic electrochemiluminescent serological immunoassays performed on the Roche Cobas (Basel, Switzerland) to detect antibody against SARS-CoV-2 nucleocapsid protein (Elecsys^®^ Anti-SARS-CoV-2 qualitative test) or spike protein (Elecsys^®^ Anti-SARS-CoV-2 S quantitative test). Both assays were done according to the manufacturer's instructions (15, 16) and results were automatically determined by the software using the electrochemiluminescence signal obtained. The assay was evaluated using the NIBSC plasma sets 20/118 and 20/130 (Supplementary Table 3).

### Flow Cytometry (FACS)-Based Assay

Transfected 293T cells (ATCC CRL-3216™) expressing SARS-CoV-2 spike protein were kind gifts from Olivier Schwartz, Institut Pasteur, Paris, France (10). Assay was performed as previously described (9, 17). Briefly, plasma samples were diluted (1:200) in 1 × PBS with 2 mM EDTA and 0.5% BSA (PBS/BSA/EDTA) and incubated with 293T-spike expressing cells (8^*^10^4^ cells/100 μl) for 30 min on ice. The cells were washed with PBS/BSA/EDTA and stained with anti-IgM PE (dilution 1:100, Biolegend) and anti-IgG Alexa FluorTM 647 (dilution 1:600, Thermo Fisher) for 30 min on ice. Cells were washed with 1 × PBS and fixed using buffer of the True Nuclear Transcription Factor Staining kit (Biolegend). After fixing, cells were washed and resuspended in 1 × PBS. Results were acquired using FACS Canto II, BD Biosciences. The gating strategy for anti-IgM or anti-IgG positive cells was based on the 293T control cells incubated with negative SARS-CoV-2 reference plasma (17). Data were reported as percentage of positive cells for anti-IgM or anti-IgG. The National Institute for Biological Standards and Control (NIBSC) Research Reagent (20/130) and panel (20/118, both WHO Solidarity II; Supplementary Table 3) was utilized to set the cutoff for positivity based on the background staining of the negative SARS-CoV-2 plasma (17) and calculated following formula: cut-off = % positive cells + 2 × standard deviation, resulting in a threshold for positivity for the IgM assay of <1.6% positive cells and for IgG <1.5% positive cells.

### Multiplex Microsphere-Based Immunoassay (MIA)

To characterize the pattern of the antibody response further, a multiplex microsphere-based immunoassay (MIA) was used previously set-up at Institut Pasteur, Paris, France. Commercially available, recombinant SARS-CoV-2 antigens (S1 and S2 proteins; The Native Antigen Company, Kidlington, UK) were coupled to MagPlex microsphere (Luminex Corp., Austin, TX, US), an approach used for other emerging pathogens before (18). Besides these antigens, antigens of SARS-CoV-1 (N and S1 proteins), MERS-CoV (S and S1 proteins), hCoV 229E (N protein), hCoV HKU1 (S1 protein) and hCoV NL63 (N protein) were used to investigate serological cross-reactivity. The MIA procedure was performed at Institut Pasteur du Cambodge as described before (18). Briefly, microspheres of all antigens were mixed with the diluted serum samples (1:400), and then incubated in the dark under constant shaking with 2 μg/mL anti-human IgG phycoerythrin-conjugated antibody (Life technologies, Carlsbad, CA, US) for 30 min at RT. Afterwards, the mean fluorescence intensity (MFI) of each microsphere set was quantified using a MagPix instrument (Luminex Corporation, Austin, TX, US). Results were expressed as relative MFI after subtracting the background MFI (microspheres without added serum). For the calibration of the SARS-CoV-2 antigens NIBSC reference plasma panels 20/118 and 20/130 (Supplementary Table 3) were used.

### Statistical Analyses

Calculations, figures and statistics were generated using Prism 9.1.2 (GraphPad Software). The data were analyzed for statistical normality before performing further statistical tests. For all analyses the significance level was α = 0.05, and *p*-values < 0.05 were considered significant. Calculation of sensitivity, specificity, as well as negative and positive predictive values were calculated as defined by Altman and Bland (19). Confidence intervals for sensitivity and specificity and accuracy are Clopper-Pearson confidence intervals. Confidence intervals for the predictive values are the standard logit confidence intervals given by Mercaldo et al. (20).

## Results

### Development and Validation of In-House IgG ELISA and PRNT

The first SARS-CoV-2 positive case in Cambodia was diagnosed on January 27th, 2020, and isolation attempts started immediately after that. Due to the long sample transfer between the patient (located in the South of Cambodia in Sihanoukville) to the laboratory on the capital city of Phnom Penh (230 km), and the late stage of viremia (low viral titer) the isolation attempts in Vero and VeroE6 cells of this imported were unsuccessful. Intensive contact tracing did not identify any contact or newly imported cases until March 7th, 2020. This time the sample transport was carried out properly on ice and the viral load in the sample was higher (ct E gene: 20.3), so the immediate isolation was successful on both cell lines Vero and VeroE6, leading to the isolate designated 1775, that was used to set up the ELISA and PRNT. Due to a higher success rate and faster viral growth, Vero cells were used for virus isolation and cultivation from then on. However, due to the more regular formation of SARS-CoV-2-induced plaques in VeroE6 cell monolayers compared to the Vero cells, VeroE6 cells were used for quantification of neutralizing antibodies by PRNT. The IgG ELISA was set-up with the same viral isolate as the PRNT. The predicated, concentrated and UV-inactivated virus was used as antigen for plate coating. Only 3 weeks after initial isolation of the SARS-CoV-2 strain 1775, both PRNT and ELISA were ready to be used for antibody quantification. However, the actual application of both in-house assays was put on hold until verification with the SARS-CoV-2 reference research reagent 20/130 and panel 20/118 acquired from the National Institute for Biological Standards and Control (NIBSC) through the WHO Solidarity II program. Our in-house PRNT results were in agreement with PRNT50 and SARS-CoV-2 pseudotyped vesicular stomatitis virus (VSV-PV) neutralization data provided by NIBSC (Table 1), although our PRNT50 titers were on average 3-fold higher than the titers achieved with the NIBSC live-virus (CPE), VSV-PV and PRNT assays. Our in-house ELISA was also 100% congruent to the EuroImmune IgG ELISA (Lübeck, Germany) and the NIBSC in-house ELISA using stabilized spike protein (B. Graham, NIAID/NIH, Bethesda, MD, USA).

**Table 1:** Results for the NIBSC reference samples.

**NIBSC plasma:**	**20/120**	**20/122**	**20/124**	**20/126**	**20/128**	**20/130**
NIBSC NT: live virus	200	70	40	35	<20	1,280
VSV-PV	267	90	20	<20	<20	2,240
PRNT50	107	33	13	<20	<20	853
NIBSC ELISA: Euroimmun IgG	POS (8.59)	POS (3.47)	POS (1.62)	Neg (0.64)	Neg (0.21)	POS (7.77)
Euroimmun IgA	POS (10.1)	POS (1.1)	POS (1.84)	POS (1.63)	Neg (0.02)	POS (9.74)
IgG S1	5,580	3,202	1,636	1,181	<50	5,388
IgG N	3,417	2,425	3,296	995	<50	17,197
IgG spike	2,693	1,488	118	8	<50	2,707
IgM	POS	POS	neg	POS	neg	POS
Anti-S IgM (% positive cells)	POS (53.62)	POS (11.85)	Neg (0)	Neg (0)	Neg (0)	POS (80.25)
Anti-S IgG (% positive cells)	POS (78.43)	POS (67.26)	POS (8.65)	POS (15.65)	Neg (0)	POS (74.41)
Anti-N ECLIA (COI)	POS (5.22)	POS (77.78)	POS (7.33)	POS (7.14)	Neg (0.097)	POS (3.98)
In-house IgG ELISA (OD_405_)	POS (1.28)	POS (1.22)	POS (1.00)	Neg (0.57)	Neg (0.70)	POS (1.30)
In-house PRNT (PRNT50)	POS (476)	POS (229)	POS (47)	Neg (<20)	Neg (<20)	POS (2,488)
MIA (MFI): SARS-CoV-2 N	POS (6,269)	POS (11,815)	POS (12,392)	POS (9,227)	Neg (446)	POS (25,980)
SARS-CoV-2 S1-His	POS (777)	POS (153)	POS (200)	Neg (59)	Neg (8)	POS (3,200)
SARS-CoV-2 S1-ScFc	POS (1,926)	POS (217)	POS (151)	Neg (48)	Neg (18)	POS (1,312)
SARS-CoV-2 S2	POS (5,993)	POS (876)	POS (1,487)	Neg (297)	Neg (54)	POS (4,436)

### Study Cohort

Overall, 250 serum samples from the SARS-CoV-2 surveillance were available for analysis. All patients were either asymptomatic (84.0%) or showed only mild clinical symptoms (16.0%) like running nose, cough and fever. Study participants were categorized into four different groups based on their SARS-CoV-2 PCR and serological results (Table 2). Individuals with SARS-CoV-2 PCR-negative swab sample at primary testing were determined as negative independent from their serology results (*n* = 16), whereas participants with PCR-positive swab sample were further divided into SARS-CoV-2 seronegative (*n* = 36), early seropositive (*n* = 8) and seropositive individuals (*n* = 190). Seronegative individuals were defined by negativity in both the FACSbased assays (IgM and IgG) and ECLIAs (N- and S-antigen based). Early seropositive participants were categorized when solely positive for anti-S IgM, while seropositive subjects were positive in either the anti-S IgG FACS assay and/or one or both ECLIAs.

**Table 2:** Study cohort characteristics and results of serological assays.

	**PCR negative**	**Seronegative**	**Early seropositive**	**Seropositive**
PCR result	Negative	Positive	Positive	Positive
Serology result	Negative	Negative	IgM positive^#^	IgG positive^##^
Number of samples	16	36	8	190
Gender (F vs. M)	3 vs. 13	5 vs. 31	1 vs. 7	20 vs. 170
Age mean (range)	28.02 (0.5–63)	34.69 (21–87)	36.25 (24–38)	36.02 (5–75)
Number of symptomatic individuals	10 (62.5%)	5 (13.9%)	0 (0.0%)	25 (13.2%)
Mean days post PCR confirmation (range)	1.00 (0–2)	4.64 (1–18)	12.75 (2–23)	13.67 (0–51)
**Number of seropositive for each assay (%;median result value)**				
Anti-S IgM FACS	2 (12.5%; 0.89)	0 (0.0%; 0.81)	8 (100%; 2.17)	117 (61.6%; 2.27)
Anti-S IgG FACS ^&^	0 (0.0%; 0.18)	0 (0.0%; 0.14)	0 (0.0%; 0.24)	145 (76.3%; 12.90)
Anti-N ECLIA	0 (0.0%, 0.09)	0 (0.0%; 0.09)	0 (0.0%; 0.11)	181^*^ (96.8%; 17.40)
Anti-S ECLIA	0 (0.0%; 0.40)	0 (0.0%; 0.40)	0 (0.0%; 0.40)	183^**^ (97.3%; 42.94)
In-house IgG ELISA	0 (0.0%; 0.36)	3^$^ (8.3%; 0.42)	2^$^ (25.0%; 0.88)	180^$^ (94.7%; 1.54)
In-house PRNT ^&^	0 (0.0%; 0.01)	0 (0.0%; 0.01)	6 (75.0%; 33.50)	173 (91.1%; 124.50)

# *FACS-based IgM assay*.

## *FACS-based IgG assay and/or ECLIAs*.

& *Samples with a result of zero were set to 0.01*.

$ *Two additional samples with equivocal result (>0.9 an < 1.0 OD_405_)*.

* *187/190 samples tested*.

** *188/190 samples tested*.

### In-House ELISA and PRNT Performance in Comparison to Commercial ECLIA

**Figure 1:**
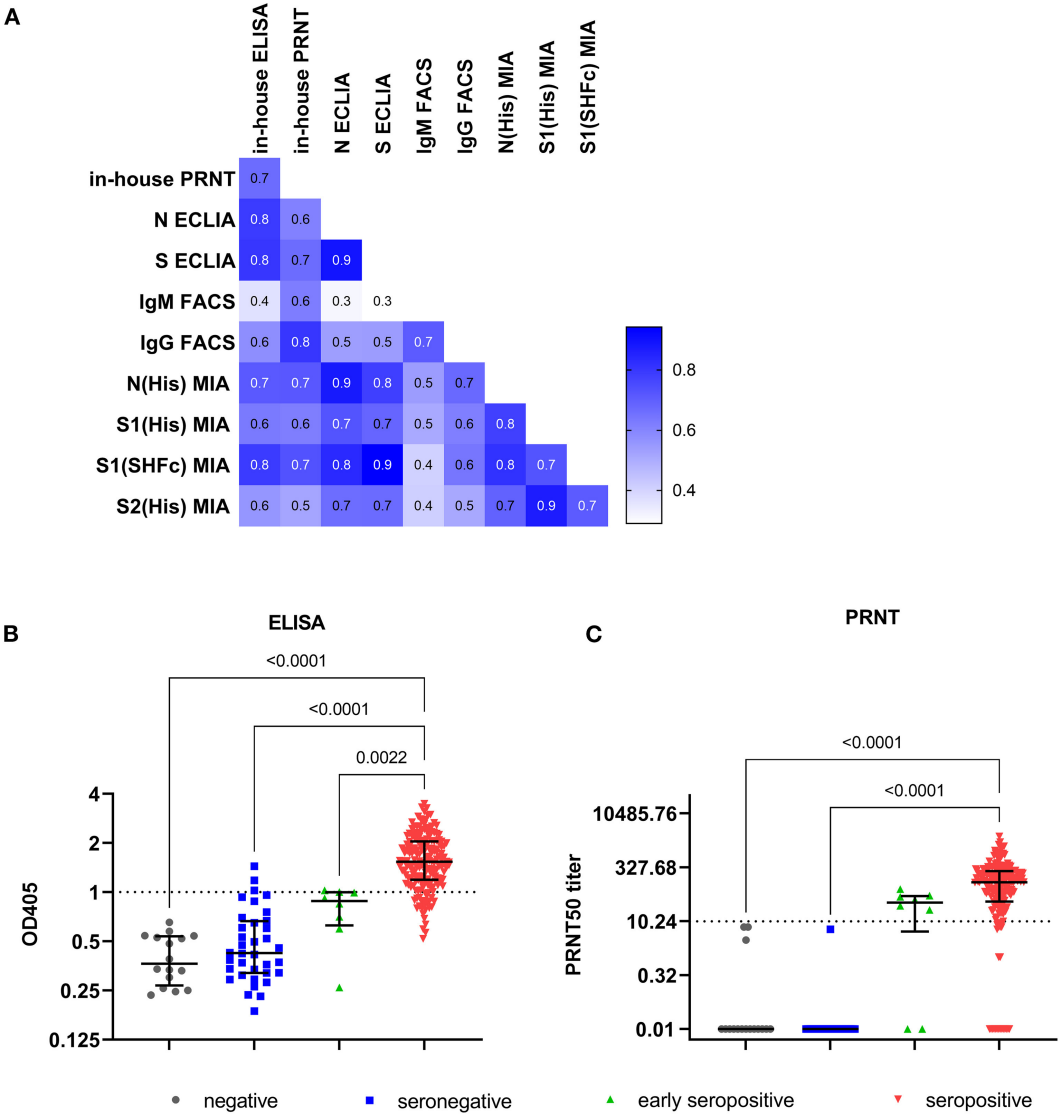
Results of in-house ELISA and PRNT. **(A)** Correlation matrix with Spearman r values of all investigated serology assay: anti-S IgM and anti-S IgG determined by flow cytometry (IgM FACS and IgG FACS, respectively), N- and S-targeting CLIA (N and S ECLIA), in-house IgG ELISA and PRNT. Individual result of each sample (total 250) for **(B)** in-house ELISA, and **(C)** in-house PRNT. Lines represent median and interquartile range. The respective thresholds (dotted line) are for in-house IgG ELISA OD_405_ ≥ 1, and for PRNT ≥ 1PRNT_50_ titer. The samples were categorized based on their SARS-CoV-2 RT-PCR result in PCR negative (n = 16; gray), and in 3 groups of SARS-CoV-2 confirmed cases: seronegative samples (n = 36; blue) with negative results in the flow cytometry based assay and ECLIAs, early seropositive samples (*n* = 8; green) that are positive for anti-S IgM, and seropositive samples (*n* = 190; red) that are positive for anti-S IgG determined by flow cytometry and/or in one or both ECLIAs. Multiple comparison was performed by Kruskal-Wallis test with α = 0.05.

The anti-S ECLIA identified more seropositive individuals (97.3%; Supplementary Figure 1A) compared to the anti-N Elecsys (96.8%; Supplementary Figure 1B). The overall comparison of results between the in-house assays and the commercial ECLIA (Figure 1A; respective *p*-values Supplementary Table 1) revealed a positive correlation between ELISA and anti-N ECLIA (Spearman r = 0.79; *p* < 0.0001) and anti-S ECLIA (Spearman r = 0.80; *p* < 0.0001), as well as between the PRNT and the anti-N ECLIA (Spearman r = 0.61; *p* < 0.0001), and anti-S ECLIA (Spearman r = 0.66; *p* < 0.0001). The classification of the sample cohort based on SARS-CoV-2 PCR, FACS and ECLIA results also showed that the PRNT was 100% specific, whereas the ELISA had a specificity of 94.2% (Table 3). The results of the in-house ELISA stratified into the different serological groups (Figure 1B) demonstrate this lower sensitivity, as three from seronegative individuals showed positive results in the in-house IgG ELISA (Table 1), indicating false positive results. Additionally, the in-house ELISA detected SARS-CoV-2 IgG antibodies in two of the eight early seropositive samples (IgM positive/IgG negative in FACS and ECLIA). Neutralizing antibodies were identified in 75% of the early seropositive study participants (Figure 1C). Furthermore, the in-house ELISA and PRNT identified 94.7 and 91.1% of the samples positive for SARS-CoV-2 antibodies, respectively. Accordingly, the sensitivity of IgG ELISA and PRNT was only slightly lower (94.7 and 91.1%, respectively; Table 3) than the best performing assay in our study, the anti-S ECLIA (97.3%).

**Table 3:** Evaluation of the serological tests.

**Value (95% CI)**	**Sensitivity**	**Specificity**	Positive predictive value^*^	Negative predictive value^*^	**Development time^#^**	**Running cost per sample^##^**
Anti-S IgM FACS	61.6% (54.3–68.5%)	96.2% (86.8–99.5%)	98.9% (95.9–99.7%)	30.6% (26.8–34.8%)	2 months	$0.5
Anti-S IgG FACS	76.3% (69.6–82.2%)	100.0% (93.2–100.0%)	100.0% (n/a)	42.7% (36.6–49.0%)	2 months	$0.5
Anti-N ECLIA	96.8% (93.2–98.8%)	100.0% (93.2–100.0%)	100.0% (n/a)	84.6% (71.5–92.4%)	4 months	$3.3
Anti-S ECLIA	97.3% (93.9–99.1%)	100.0% (93.2%−100.0%)	100.0% (n/a)	86.9% (73.7–94.0%)	10 months	$7.3
In-house IgG ELISA	94.7% (90.5–97.5%)	94.2% (84.1–98.8%)	98.9% (96.9–99.6%)	76.0% (63.3–85.3%)	3 months	$0.3
PRNT	91.1% (86.1–94.7%)	100.0% (93.2–100.0%)	100.0% (n/a)	66.4% (55.6–75.6%)	2 months	$2.5

* *Calculated for a seroprevalence of 85%*.

# *Since WHO declaration on Public Health Emergency of International Concern (PHEIC on January 30th, 2020)*.

## *Excluding equipment acquisition, personnel, and facility costs*.

### Performance of In-House FACS-Based Immunoassay

Eight individuals of the whole study cohort were solely positive for anti-S IgM antibodies, classifying them as early seropositive. However, more than half of the seropositive individuals (55.8%, 106/190) had both IgM and IgG antibodies binding to the S antigen expressed on the surface of cells measured by FACS (Supplementary Figures 1A,B, respectively). The titers for S-binding IgM (expressed as % of cells with bound antibodies) were not significantly different between the seropositive and the early seropositive group (*p* > 0.9999; Supplementary Figure 1C). In contrast, anti-S IgG levels detected by FACS (Supplementary Figure 1D) are significant lower in the early seropositive individuals (median: 0.24%) compared to the seropositive study participants (median: 12.90%; *p* = 0.0082). Interestingly, two (12.5%) of the 16 PCR-negative individuals displayed IgM antibodies against SARS-CoV-2. Taken together, the anti-S FACS assays had a sensitivity of 61.6 and 76.3% for IgM and IgG, respectively. The anti-S IgG FACS assay was 100% specific, whereas the anti-S IgM FACS assay had a specificity of 96.2%. In comparison to the other in-house assays, the IgG ELISA and PRNT performed better in terms of sensitivity (Table 3). However, the FACS was developed as fast as the PRNT and is similarly cheap as the in-house ELISA. Also, the results achieved with the anti-S IgG FACS correlated positively with the in-house IgG ELISA (Spearman r = 0.58; *p* < 0.0001; Figure 1A) and even better with the PRNT (Spearman r = 0.80; *p* < 0.0001). Furthermore, the anti-S IgM FACS also correlates with the PRNT (Spearman r = 0.62; *p* < 0.0001).

### Performance of SARS-CoV-2 MIA

Nearly all study participants (248/250; Supplementary Table 2) were additionally tested for binding antibodies to multiple coronavirus antigens by the multiplex microsphere-based immunoassay (MIA; Table 4). First, the threshold for the SARS-CoV-2 antigens was calibrated with the NIBSC reference samples (Table 1). The detection of anti-N IgG by MIA even allowed the positive identification of NIBSC reference 20/126 that lead to equivocal results across diverse IgG assays. Afterwards we determined the IgG response of our study cohort to SARS-CoV-2 N, S1 and two formulations of S2 antigens (Supplementary Figure 2). The majority of the formerly classified seropositive samples (98.9%) had antibodies binding to the viral N protein (Supplementary Figure 2A). Remarkably, a number of the other study participants also had N-binding antibodies: 50.0% (8/16) of the SARS-CoV-2 negative individuals, 66.7% (24/36) of seronegative individuals, and 87.5% (7/8) of the early seropositive individuals. Nearly all seropositive individuals also had antibodies binding viral S2 domain (89.4%, 168/188, Supplementary Figure 2B) and/or S1 domain (S1-SHFc: 90.9%, 169/186, Supplementary Figure 2C; S1-His: 94.6%, 176/186, Supplementary Figure 2D). For the SARS-CoV-2 negative individuals, only one of the SARS-CoV-2 negative individuals (1/16) also had antibodies against SARS-CoV-2 S1 SHFc-tagged (not for the respective His-tagged antigen). Among seronegative study participants some of them also displayed antibodies targeted against S2 (19.4%, 7/36) and a few against S1 (His–tagged: 3/36; SHFc-tagged antigen: 2/36). The majority of the early seropositive individuals had antibodies against S2 (87.5%, 7/8) and S1 His-tagged antigen (62.5%, 5/8), whereas only two were detected with antibodies against S1 SHFc tagged. The results of the MIA for the SARS-CoV-2 antigens correlated positively with the results of the formerly investigated serological assays (flow cytometry based assays, ECLIAs, ELISA and PRNT; Figure 1A). The in-house assays correlated best with the anti-N MIA (IgG FACS, ELISA PRNT Spearman r = 0.67, 0.71, and 0.72, respectively; *p* <0.0001; Figure 1A). Besides this, the positive correlation between these in-house assays was the strongest toward the S1 (SHFc) antigen (IgG FACS, ELISA PRNT Spearman r = 0.64, 0.79, and 0.73, respectively; *p* <0.0001).

**Table 4:** Results of multiplex antigen serological testing.

**Median relative MFI (95% CI)**	**PCR negative**	**Seronegative**	**Early seropositive**	**Seropositive**
SARS-CoV-2 N^&^	939 (742–1,563)	1,235 (989–1,730)	1,632 (869–2,554)	10,500 (9,285–11,550)
SARS-CoV-2 S1^&^	29 (21–46)	38 (31–50)	181 (33–395)	355 (316–459)
SARS-CoV-2 S1^*^	51 (37–59)	47 (38–63)	85 (22–124)	402 (318–510)
SARS-CoV-2 S2^&^	82 (26–218)	166 (90–290)	743 (83–1,226)	1,797 (1,535–2,163)
SARS-CoV-1 N^&^	139 (77–295)	372 (154–458)	175 (51–313)	6,100 (5,279–6,768)
SARS-CoV-1 S1^&^	1,491 (289–3,497)	917 (575–1,215)	1,313 (557–1,531)	1,169 (1,034–1,474)
MERS-CoV S1^&^	50 (41–69)	53 (47–76)	90 (42–116)	56 (54–61)
MERS-CoV S1 + S2^&^	1,129 (389–2,074)	940 (739–1,244)	1,048 (832–1,240)	1,478 (1,291–1,646)
hCoV HKU1 S^&^	3,442 (31–6,447)	4,505 (3,040–5,845)	8,516 (8,206–9,124)	6,108 (5,582–7,046)
hCoV NL63 N^&^	481 (198–905)	574 (391–1,246)	1,832 (986–2,098)	568 (493–632)
hCoV 229E N^&^ (4 μg)	979 (591–1,325)	999 (839–1,306)	1,319 (1,074–1,962)	1,220 (1,163–1,367)
hCoV 229E N^&^ (10 μg)	566 (425–741)	604 (508–797)	876 (690–1,267)	672 (632–745)

& *His-tagged antigen*.

* *SHFc-tagged antigen*.

### IgG Response in Early Convalescent Serum of COVID-19 Patients to Other Coronaviruses

For the antigens against coronaviruses other than SARS-CoV-2, we were not able to achieve suitable positive and negative serological controls, and therefore could not classify results into negative and positive. However, comparing the results of the different patient groups in our cohort allowed the determination of certain cross-reactivities of SARS-CoV-2 immune response to other highly pathogenic beta-coronaviruses (Supplementary Figure 3) and seasonal human coronaviruses (Supplementary Figure 4). SARS-CoV-2 seropositive individuals had a significantly higher response to the N protein of SARS-CoV-1 (*p* < 0.0001; Supplementary Figure 3A), but not against the S1 antigen of SARS-CoV-1 (Supplementary Figure 3B) and MERS-CoV Supplementary Figure 3C, as well as the whole S antigen (S1 + S2) of MERS-CoV (Supplementary Figure 3D). Furthermore, Spearman correlation revealed a positive correlation for the formerly evaluated serological assays with the MIA using SARS-CoV-1 antigen, but not for the antigens of the other highly pathogenic beta-coronaviruses. However, the reactivity against SARS-CoV-1 S1 antigen correlated with the antibodies found against MERS-CoV S1 + S2 antigen (Supplementary Table 1).

Furthermore, we observed a notable amount of antibodies binding S1 of seasonal human beta-coronavirus HKU1 (Supplementary Figure 4A) in the seropositive and early seropositive individuals indicated by significantly higher MFI signals in these two groups (r = 0.028 and r = 0.020, respectively). The early seropositive individuals had a significant higher MFI signal to hCoV NL63 N protein (Supplementary Figure 4B) than all other study participants including the seropositive individuals. We found a marginal negative correlation of the MIA for hCoV NL63 N against FACS-based assays, ECLIAs, ELISA, PRNT, and MIA for SARS-CoV-2 antigens as well as SARS-CoV-1 N antigen (Supplementary Table 1; Spearman r ranging from −0.221 to −0.044). The binding capacity for the N antigen of hCoV 229E was inconclusive: when the microspheres were coated with 4 μg of the antigen we measured lower binding capacity among the seropositive individuals (Supplementary Figure 4C), whereas with an increased antigen load (10 μg) the seropositive individuals had on average a higher binding reaction (Supplementary Figure 4D).

## Discussion

The continuing COVID-19 pandemic combined with increased vaccination efforts (including homologous and heterologous boosters) puts high demand on serological assays for SARS-CoV-2 antibodies. The range of available tests vary from fully automated, clinical immunoassays and commercial bench-top ELISAs, to in-house solutions developed by individual institutions. Early in the pandemic, due to paucity of commercial regents, there was a critical need for serological assays using country-specific isolates. Therefore, we developed both an in-house ELISA and live-virus neutralization test (PRNT). The in-house IgG ELSIA had a slightly higher sensitivity than the PRNT, however, the PRNT was 100% specific whereas the ELISA produced some false-positive results. Following the availability of commercial ECLIAs, we find the PRNT is as specific as both commercial tests; however, its sensitivity lay between them. In addition, we found the sensitivity of the anti-N ECLIA similar than stated by the manufacturer (7–13 days post PCR confirmation 85.3%; ≥14 days 99.5%) (15), whereas for the anti-S ECLIA we observed a slightly higher sensitivity (7–13 days post PCR confirmation 85.5%; 14–20 days 89.2%) (16) with our sample cohort (13.8 days post PCR confirmation).

The in-house IgG ELISA also performed nearly as well as both ECLIAs. In contrast to many in-house and commercially available ELISAs (3, 21), we did not use recombinant S or N antigens but full virus, harvested from virus culture supernatant and inactivated by UV light. This option for producing SARS-CoV-2 antigen allows laboratories without the ability to produce or purchase recombinant antigens the set-up of a suitable ELISA if they have the capacity to handle live SARS-CoV-2 or can obtained inactivated virus from a reference laboratory. Interestingly, we found anti-SARS-CoV-2 IgG antibodies in 8% of seronegative study participants and in 25% of early seropositive individuals using the in-house IgG ELISA. This reactivity could be either due to cross-reactivity from previous hCoV infection(s) (22) or because the whole virus presents additional antigenic SARS-CoV-2 proteins that might be targeted preferentially earlier in the immune response (envelope protein E, membrane protein M) (23, 24).

Besides of their comparable performance to the commercial immunoassays, both in-house IgG ELISA and PRNT were developed rapidly, within 3 weeks after isolation of the first Wuhan-like SARS-CoV-2. Despite being located in a Least Developed Country (LDC), our laboratory has a broad serological routine testing capacity, and we were able to set-up these assays independently of the delivery of SARS-CoV-2-specific reagents like recombinant antigens or virus-specific antibodies. The sole isolation, and production of the virus was enough for the implementation of these methods. However, as SARS-CoV-2 has to be handled *in vitro* under biosafety level 3 (BSL3) conditions, the existence of such a facility is an irrevocable necessity. Neglecting the costs and difficulties of running a BSL3 laboratory, especially in an LDC, and global variability in staff costs, these assays are relatively inexpensive compared to the commercial platform immunoassays (Table 3). Furthermore, isolation of virus allows for in-house standardization of the serological results. In general, establishment of universal standard references lags far behind the rapid development of serological in-house methods. Indeed, the NIBSC references used in this study were a first attempt at standardization. Equal references are available now by the US National Cancer Institute (NCI) Serological Sciences Network (SeroNet) and the WHO standard 20/136 (replacing the here used NIBSC standard 20/130) (25). Due to the delay in establishing the WHO standard and sample availability in our cohort we were not able to evaluate our in-house assays with the new WHO standard. Further work in our lab and others will utilize these new standards for unified and comparable reporting of the measured antibody response, e.g., in the arbitrary unit of binding antibody units (BAU)/mL, and should be made readily available to laboratories around the world.

In addition to the in-house assays and commercial ECLIAs, utilization and comparison of the flow cytometry based assay allowed the separate detection of IgM and IgG antibodies binding to S-expressing cells, which enables generally the discrimination between recent viral infection (IgM positive/IgG negative) and past infections (IgM positive/IgG positive or IgM negative/IgG positive). However, in the case of SARS-CoV-2, the utility of IgM testing remains dubious due to the reporting of nearly simultaneous IgM and IgG response (26–28). Indeed, our PRNT shows a neutralization capacity in 75% of solely IgM positive study participants (early seropositive), suggesting neutralizing capacity of IgM antibodies toward SARS-CoV-2, similar to what has been observed with purified IgM from convalescent COVID-19 patients (29).

Further, comparison the multiplex microsphere-based assay (MIA) enabled thedetection of potentially cross-reactivity between SARS-CoV-1 and−2 anti-N anti-N antibodies. In contrast, the sera of SARS-CoV-2 infected patients showed no significant anti-S cross-reactivity toward the other highly pathogen betacoronaviruses SARS-CoV-1 and MERS-CoV. MIA showed cross reactivity of antibodies from SARS-CoV-2-infected individuals to the spike protein of another human beta-coronavirus, hCoV HKU1, which cannot be concluded from current in-house or commercial assays. Cross-reactivity among beta-coronaviruses were reported previously (22). Due to antigenic similarities among these beta-coronaviruses, a SARS-CoV-2 infection results in the production of cross-reactive SARS-CoV-2 antibodies as well as to an upregulation of cross-reactive hCoV antibodies (30). As previous infection with hCoVs in our cohort cannot be determined, this finding could be associated with former exposure to HKU1, or other confounding factors. However, similar results were observed in children with COVID-19 where hCoV-HKU1 S-binding antibodies strongly correlated with SARS-CoV-2 antibodies (31).

While showing good performance of in-house assays against commercial test, this study does have several limitations. Our assessment of serological assay performance relies on asymptomatic and mild SARS-CoV-2 infected patients. For the latter, we were able to determine the period between serum sampling and PCR confirmation, but time since onset of symptoms. Further, another limitation was that we had no access to pre-pandemic samples for the purpose of the study, and the opportunistic sampling revealed only a very limited number of SARS-CoV-2-negative individuals that we could include in our study. In addition, the cohort is skewed toward an over-representation of males (88.4%). Furthermore, the comparison of serological assays that differ not only greatly in their applied antigen(s) but also the antibody class they determine, should be taken cautiously, especially as we determined not only binding antibodies abut also functional neutralizing antibodies, and the used antigens derived from different virus strains and were used in various formulations.

In terms of other assay limitations, MIA results were influenced by the amount of antibodies bound to microsphere, as different amounts of the same hCoV 229E antigen used for the coating of the microspheres lead to contrary results. More antigen used for the coating did not only resulted in overall higher relative MFI signals but also a specific response observed for the SARS-CoV-2 infected patients. With the low antigen amount, all study participants (including the SARS-CoV-2 negative ones) showed similar binding capacities, which is not surprising as a certain prevalence is expected for this seasonal hCoV (22, 30). Additionally, the tag of the antigen also influenced the reactivity to potential binding antibodies, as we identified more individuals with SARS-CoV-2 S1 –binding antibodies with the His-tagged antigen than with the SHFc-tagged antigen (Supplementary Figures 2C,D). The observed reactivity could be due to a reaction to the histidine-tag rather than to the SARS-CoV-2 antigen itself, as some pathogens like *Plasmodium falciparum* (32) have histidine-rich epitopes and therefore former infections might have led to anti-histidine antibodies in these individuals.

Overall, our results demonstrate that in-house serological assays, when developed, calibrated, and evaluated correctly, perform nearly as well as commercial assays. This confirmation is critical for early introduction, outbreak control, and tracing efforts, as these assays can be developed and deployed very quickly following initial virus isolation and without the necessity of purchasing virus-specific reagents. Therefore, in-house, tailored diagnostic solutions are a viable and advantageous solution, especially in resource-limited countries experienced laboratories. These alternatives are especially critical when commercial assays are not available due to global development or supply issues, or when a sophisticated, expensive automation system, such as the Roche Cobas, is lacking. Additionally, these assays can quickly be adapted for new variants or new viruses, greatly increasing diagnostic capacity ahead of commercial development. Hence, in-house serological assays can serve as key factors in seroprevalence investigations and guiding public health measures.

## Data Availability Statement

The original contributions presented in the study are included in the article/Supplementary Material, further inquiries can be directed to the corresponding author/s.

## Author Contributions

HA, TC, VD, and EK contributed to conception and design of the study. HA, JV, and PD acquired the necessary funding. Data acquisition and analysis was performed by HA, CE, SL, SI, SE, HV, CS, SC, and GD. HA and CE wrote the first draft of the manuscript. All authors contributed to manuscript revision, read, and approved the submitted version.

## Funding

This work was supported by the *URGENCE COVID-19* fundraising campaign of Institut Pasteur. HA was supported by the German Centre for International Migration and Development (CIM). This work has been supported by WHO Solidarity II, global serologic study for COVID-19, with funding from the German Federal Ministry of Health (BMG) COVID-19 Research and development to WHO. The 293T cells with stable expression of SARS-CoV-2 spike were kind gifts from Timothée Bruel and Olivier Schwartz, Institut Pasteur, Paris, France.

## Conflict of Interest

The authors declare that the research was conducted in the absence of any commercial or financial relationships that could be construed as a potential conflict of interest.

## Publisher's Note

All claims expressed in this article are solely those of the authors and do not necessarily represent those of their affiliated organizations, or those of the publisher, the editors and the reviewers. Any product that may be evaluated in this article, or claim that may be made by its manufacturer, is not guaranteed or endorsed by the publisher.
