# Supplementary material for: Rapid Generation of In-House Serological Assays Is Comparable to Commercial Kits Critical for Early Response to Pandemics: A Case With SARS-CoV-2
Source: Front Med (Lausanne). 2022 May 6;9:864972. doi: 10.3389/fmed.2022.864972 (PMC9121123; doi:10.3389/fmed.2022.864972)
Supplement: Supplementary file 1 [file Data_Sheet_1.PDF]

Supplemental material

Supplementary Table 1: Spearman correlation p values

	IgM SFlow	IgG SFlow	N Elecsys	S Elecsys	ELISA	PRNT	MIA SARS-CoV-2 N	MIA SARS-CoV-2 S1 (His)	MIA SARS-CoV-2 S1 (SHFc)	MIA SARS-CoV-2 S2	MIA SARS-CoV-1 N	MIA SARS-CoV-1 S1	MIA MERS-CoV S1	MIA MERS-CoV S1+S2	MIA hCoV NL63 N	MIA hCoV 229E N (4ug)	MIA hCoV 229E N (10ug)	MIA hCoV HKU1 S1
**IgM SFlow**		****	****	****	****	****	****	****	****	****	****	0.5043	0.0456	0.4807	0.0141	0.8329	0.7755	0.0276
**IgG SFlow**	****		****	****	****	****	****	****	****	****	****	0.4629	0.3683	0.1247	0.0432	0.3144	0.9638	0.9941
**N Elecsys**	****	****		****	****	****	****	****	****	****	****	0.0161	0.1041	0.0018	0.3710	0.0022	0.2005	0.0068
**S Elecsys**	****	****	****		****	****	****	****	****	****	****	0.0112	0.2135	0.0419	0.2449	0.0031	0.2235	0.1202
**ELISA**	****	****	****	****		****	****	****	****	****	****	0.0327	0.4522	0.0126	0.1535	0.2174	0.6236	0.3265
**PRNT**	****	****	****	****	****		****	****	****	****	****	0.5503	0.5855	0.3106	0.0005	0.7642	0.7073	0.7623
**MIA SARS-CoV-2 N**	****	****	****	****	****	****		****	****	****	****	0.0254	0.0239	0.0009	0.1928	0.0028	0.1550	0.0349
**MIA SARS-CoV-2 S1 (His)**	****	****	****	****	****	****	****		****	****	****	0.3091	0.0069	0.0142	0.3734	0.0051	0.1324	0.0009
**MIA SARS-CoV-2 S1 (SHFc)**	****	****	****	****	****	****	****	****		****	****	0.0033	0.0004	0.0093	0.1832	0.0003	0.0484	0.0283
**MIA SARS-CoV-2 S2**	****	****	****	****	****	****	****	****	****		****	0.0480	0.0012	0.0319	0.4905	0.0601	0.0927	****
**MIA SARS-CoV-1 N**	****	****	****	****	****	****	****	****	****	****		0.0618	0.0600	0.0020	0.0597	0.0216	0.4802	0.0168
**MIA SARS-CoV-1 S1**	0.5043	0.4629	0.0161	0.0112	0.0327	0.5503	0.0254	0.3091	0.0033	0.0480	0.0618		****	****	****	****	****	0.1500
**MIA MERS-CoV S1**	0.0456	0.3683	0.1041	0.2135	0.4522	0.5855	0.0239	0.0069	0.0004	0.0012	0.0600	****		****	0.0528	****	****	0.0053
**MIA MERS-CoV S1+S2**	0.4807	0.1247	0.0018	0.0419	0.0126	0.3106	0.0009	0.0142	0.0093	0.0319	0.0020	****	****		****	****	****	0.5004

	IgM SFlow	IgG SFlow	N Elecsys	S Elecsys	ELISA	PRNT	MIA SARS-CoV-2 N	MIA SARS-CoV-2 S1 (His)	MIA SARS-CoV-2 S1 (SHFc)	MIA SARS-CoV-2 S2	MIA SARS-CoV-1 N	MIA SARS-CoV-1 S1	MIA MERS-CoV S1	MIA MERS-CoV S1+S2	MIA hCoV NL63 N	MIA hCoV 229E N (4ug)	MIA hCoV 229E N (10ug)	MIA hCoV HKU1 S1
**MIA hCoV NL63 N**	0.0141	0.0432	0.3710	0.2449	0.1535	0.0005	0.1928	0.3734	0.1832	0.4905	0.0597	****	0.0528	****		****	****	0.0181
**MIA hCoV 229E N (4ug)**	0.8329	0.3144	0.0022	0.0031	0.2174	0.7642	0.0028	0.0051	0.0003	0.0601	0.0216	****	****	****	****		****	0.1929
**MIA hCoV 229E N (10ug)**	0.7755	0.9638	0.2005	0.2235	0.6236	0.7073	0.1550	0.1324	0.0484	0.0927	0.4802	****	****	****	****	****		0.0254
**MIA hCoV HKU1 S1**	0.0276	0.9941	0.0068	0.1202	0.3265	0.7623	0.0349	0.0009	0.0283	0.0000	0.0168	0.1500	0.0053	0.5004	0.0181	0.1929	0.0254	

**** p<0.0001

Supplementary Table 2: Study cohort characteristics for multiplex antigen serological testing

	**PCR negative**	**Seronegative**	**Early seropositive**	**Seropositive**
PCR result	Negative	Positive	Positive	Positive
Serology result	Negative	Negative	IgM positive #	IgG positive ##
Number of samples	16	36	8	188
Gender (F vs M)	3 vs 13	5 vs 31	1 vs 7	20 vs 168
Age mean (range)	28.02 (0.5 – 63)	34.69 (21 – 87)	36.25 (24 – 38)	36.1 (5 – 70))
Number of symptomatic cases	10 (62.5%)	5 (13.9%)	0 (0.0%)	25 (13.3%)
Mean days post PCR confirmation (range)	1.00 (0 – 2)	4.64 (1 – 18)	12.75 (2 – 23)	13.7 (0 – 51)

flow cytometry based assay; ## flow cytometry based assay and/or Elecsys;

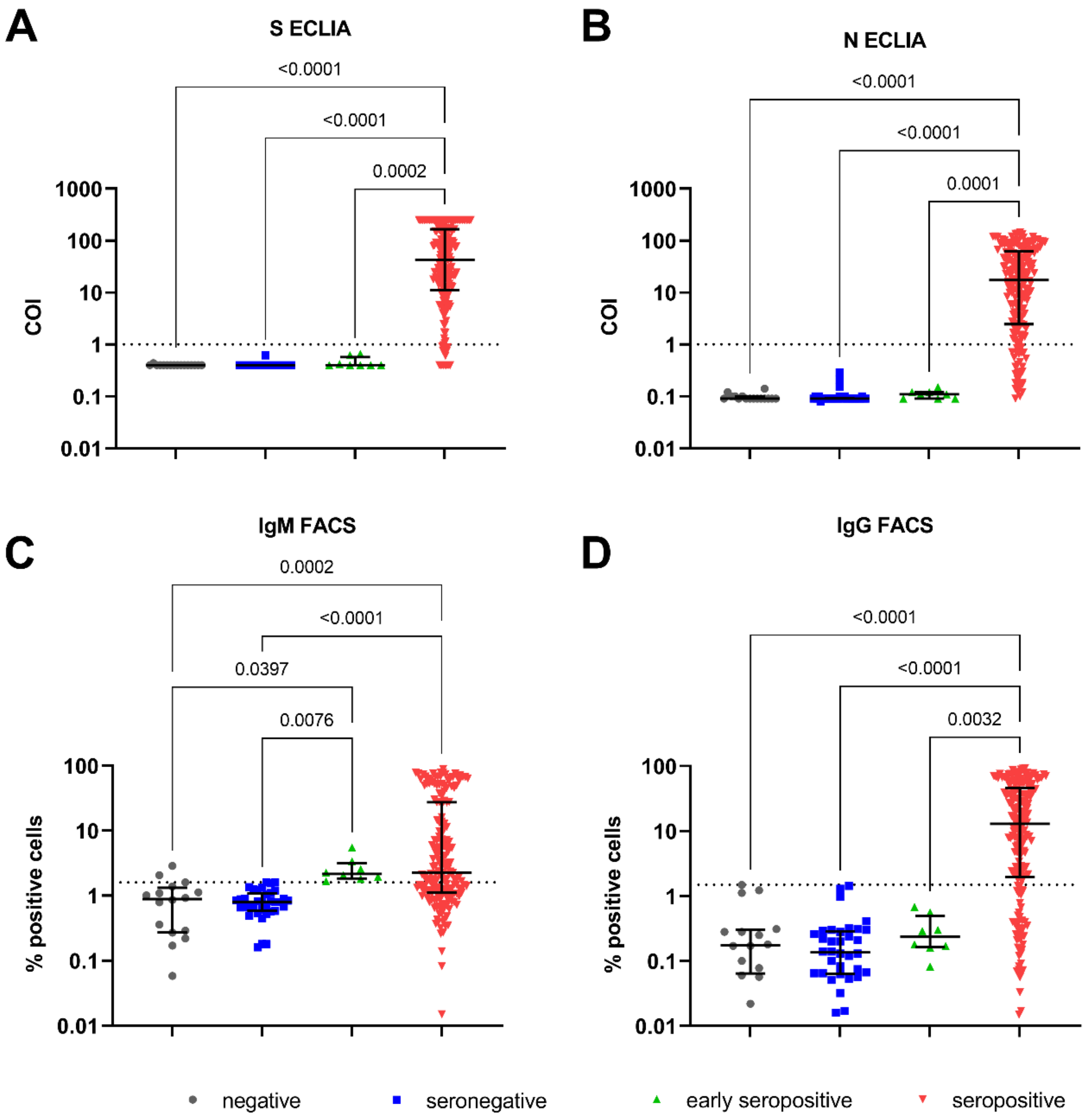

Supplementary Figure 1: Individual results of ECLIAs and FACS assays. The results of (A) anti-S ECLIA, (B) anti-N ECLIA, (C) anti-S IgM FACS, (D) anti-S IgG FACS were plotted for each sample (total 250). Lines represent median and interquartile range. The respective thresholds (dotted line) for anti-S IgM FACS > 1.5% of positive cells, for anti-S IgG FACS > 1.6% of positive cells, for ECLIAs ≥ 1 COI. The samples were categorized based on their SARS-CoV-2 RT-PCR result in PCR negative (n=16; grey) , and in 3 groups of SARS-CoV-2 confirmed cases: seronegative samples (n=36; blue) with negative results in FACS and ECLIAs, early seropositive samples (n=8; green) that are positive for anti-S IgM, and seropositive samples (n=190; red) that are positive for anti-S IgG determined by FACS and/or in one or both ECLIAs. Multiple comparison was performed by Kruskal-Wallis test with α=0.05.

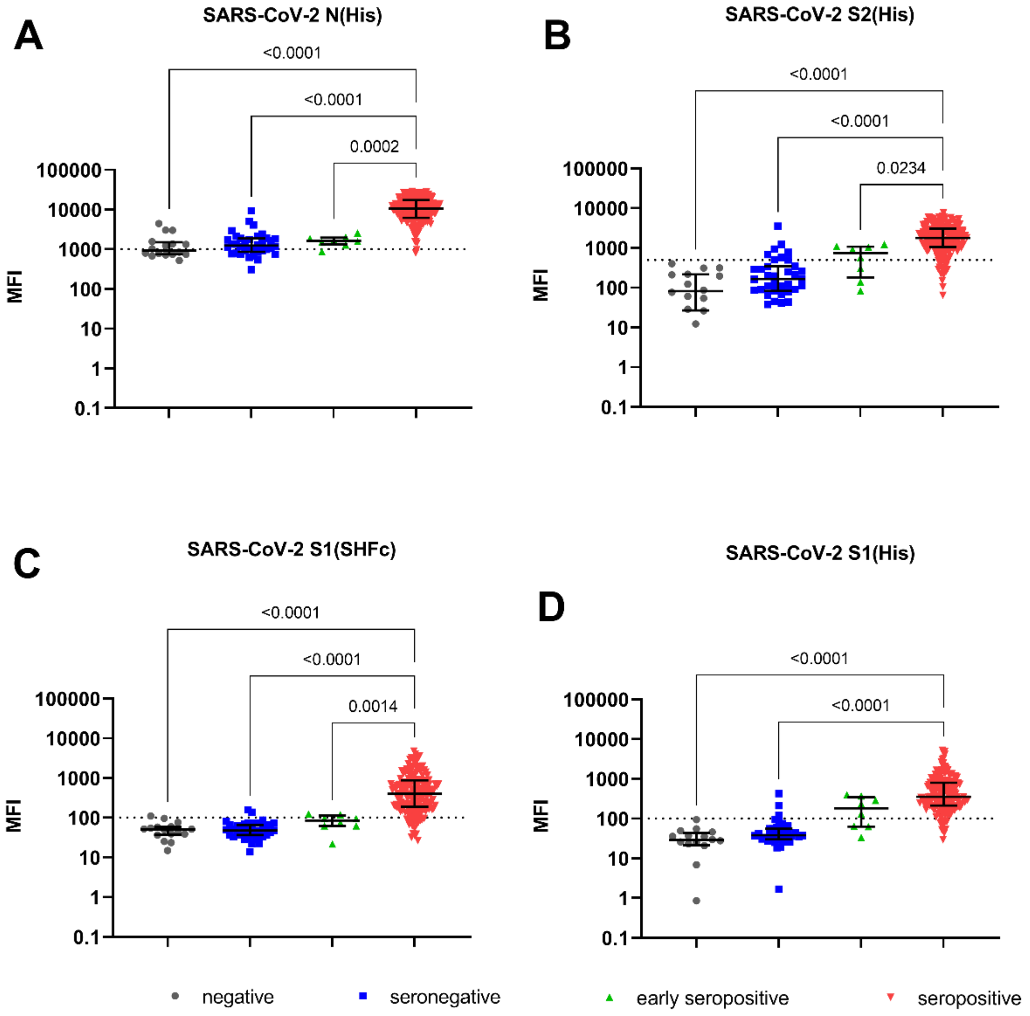

Supplementary Figure 2: Individual results for SARS-CoV-2 multiplex serology assay. The results of the microsphere-based immunoassay for recombinant SARS-CoV-2 antigens: (A) nucleocapsid (His-tagged), (B) spike S2 domain (His-tagged), (C) spike S1 antigen (SHFc-tagged), and (D) spike S1 antigen (His-tagged). Individual results are expressed in relative mean fluorescence intensity (MFI) for each sample (total 248). Lines represent median and interquartile range. The respective thresholds (dotted line) were for N-targeting MIA ≥ 1000 MFI, for S2-targetting MIA ≥ 500 MFI, and for S1-targetting MIAs ≥ 100 MFI. The samples were categorized based on their SARS-CoV-2 PCR result in PCR negative (n=16; grey) , and in 3 groups of SARS-CoV-2 confirmed cases: seronegative samples (n=36; blue) with negative results in the flow cytometry based assay and Elecsys immunoassays, early seropositive samples (n=8; green) that are positive for anti-S IgM, and seropositive samples (n=190; red) that are positive for anti-S IgG determined by flow cytometry and/or in one or both Elecsys immunoassays. Multiple comparison was performed by Kruskal-Wallis test with α=0.05.

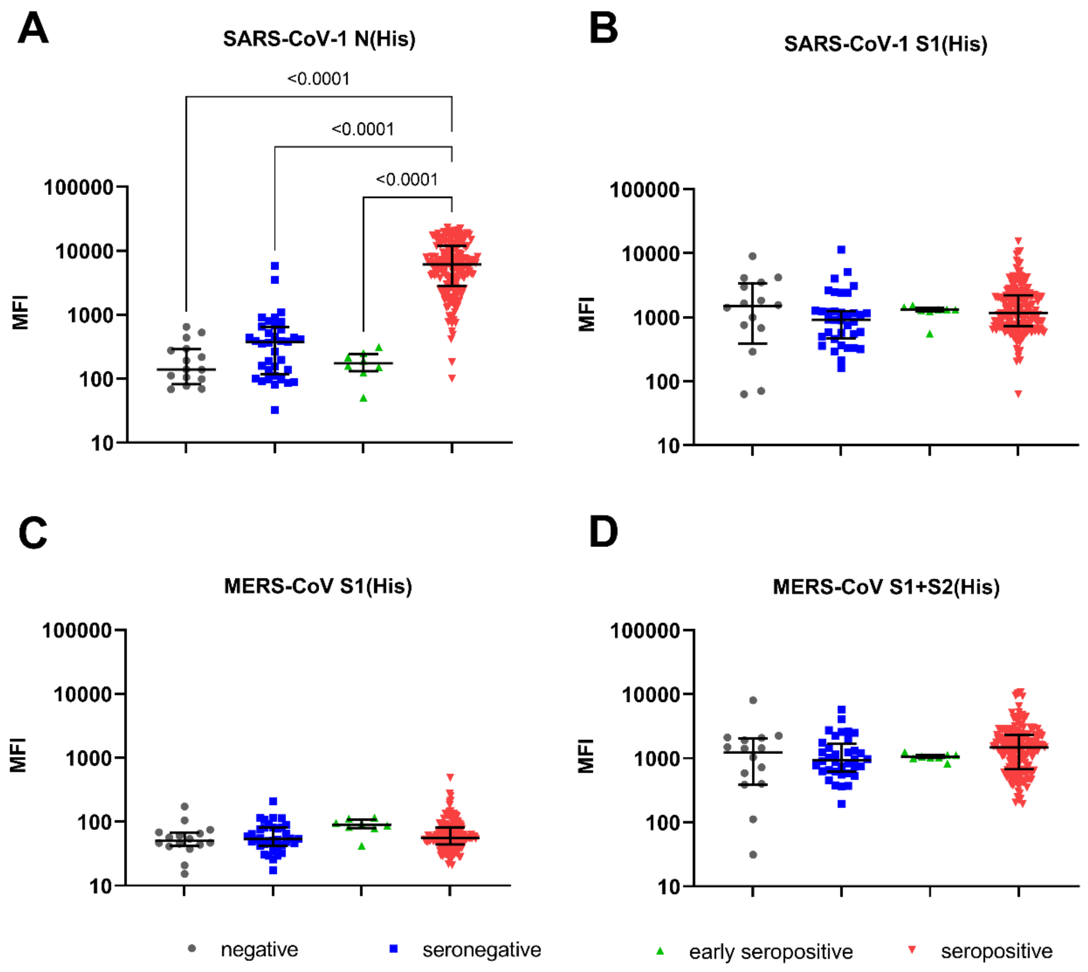

Supplementary Figure 3: Individual results for SARS-CoV-1 and MERS-CoV multiplex serology assay. The results of the microsphere-based immunoassay for recombinant SARS-CoV-1 (A) nucleocapsid, and (B) spike S1 domain), as well as MERS-CoV (C) spike S1 domain, and (D) full spike antigen (S1+S2). Individual results are expressed in relative mean fluorescence intensity (MFI) for each sample (total 248). Lines represent median and interquartile range. The samples were categorized based on their SARS-CoV-2 PCR result in PCR negative (n=16; grey) and in 3 groups of SARS-CoV-2 confirmed cases: seronegative samples (n=36; blue) with negative results in the flow cytometry based assay and Elecsys immunoassays, early seropositive samples (n=8; green) that are positive for anti-S IgM, and seropositive samples (n=190; red) that are positive for anti-S IgG determined by flow cytometry and/or in one or both Elecsys immunoassays. Multiple comparison was performed by Kruskal-Wallis test with α=0.05. Lines represent median and interquartile range.

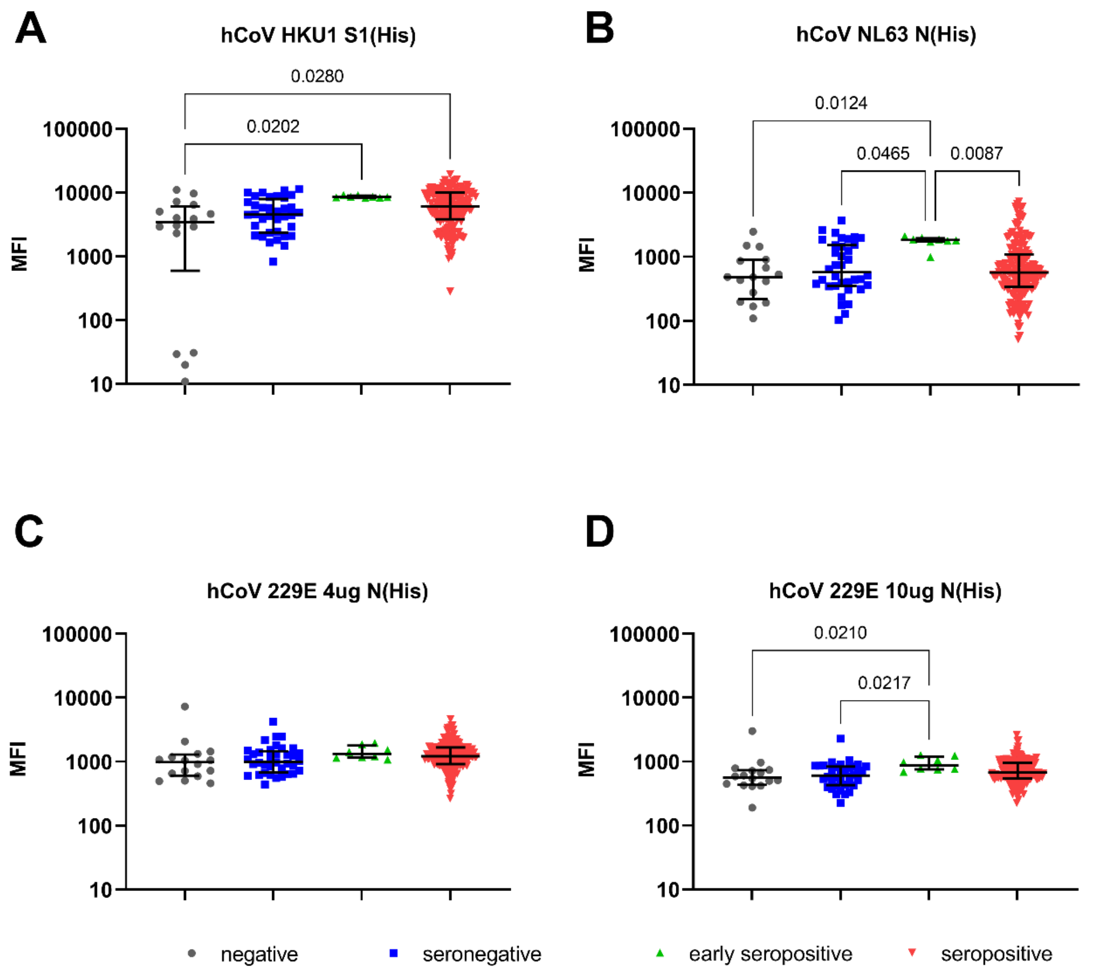

Supplementary Figure 4: Individual results for hCoVs multiplex serology assay. The results of the microsphere-based immunoassay for recombinant hCoV antigens: (A) HKU spike S1 domain, (B) NL63 nucleocapsid, (C) 229E 4 µg nucleocapsid, and (D) 229E 10 µg nucleocapsid. Individual results are expressed in relative mean fluorescence intensity (MFI) for each sample (total 248). Lines represent median and interquartile range. The samples were categorized based on their SARS-CoV-2 PCR result in PCR negative (n=16; grey) and in 3 groups of SARS-CoV-2 confirmed cases: seronegative samples (n=36; blue) with negative results in the flow cytometry based assay and Elecsys immunoassays, early seropositive samples (n=8; green) that are positive for anti-S IgM, and seropositive samples (n=190; red) that are positive for anti-S IgG determined by flow cytometry and/or in one or both Elecsys immunoassays. Multiple comparison was performed by Kruskal-Wallis test with α=0.05. Lines represent median and interquartile range.
